# Exercise-Induced Oxygen Desaturation and Cognitive Performance in Patients with Parkinson’s Disease: A Prospective Observational Study

**DOI:** 10.3390/jcm15020899

**Published:** 2026-01-22

**Authors:** Alexandra-Cristiana Gache, Elena Danteș, Andreea-Cristina Postu, Denisa-Gabriela Ion-Andrei, Adina-Milena Man, Nicoleta-Larisa Șerban, Irene Rășanu, Any Axelerad

**Affiliations:** 1 Department of Pneumology, Faculty of Medicine, Campus—Corp B, Ovidius University of Constanta, 1 University Alley, 900470 Constanta, Romania; alexandra.belu@365.univ-ovidius.ro (A.-C.G.); postu.andreea@gmail.com (A.-C.P.); denisagabriela94@gmail.com (D.-G.I.-A.); 2 Medical Doctoral School, Faculty of Medicine, Campus—Corp B, Ovidius University of Constanta, 1 University Alley, 900470 Constanta, Romania; irenedamian@yahoo.com (I.R.); docuaxi@yahoo.com (A.A.); 3 Clinical Hospital of Pneumopthisiology Constanta, 40 Santinelei Street, 900002 Constanta, Romania; 4 Department of Pneumology, “Iuliu Hatieganu” University of Medicine and Pharmacy, 400012 Cluj-Napoca, Romania; manmilena50@yahoo.com; 5 Department of Neurosurgery, Cluj County Clinical Emergency Hospital, 400347 Cluj-Napoca, Romania; larisa_serban@yahoo.com; 6 Department of Neurosciences, “Iuliu Hatieganu” University of Medicine and Pharmacy, 400012 Cluj-Napoca, Romania; 7 Clinical CF Hospital Constanta, 1 Mai 3-5 Boulevard, 900123 Constanta, Romania; 8 Department of Neurology, Faculty of Medicine, Campus—Corp B, Ovidius University of Constanta, 1 University Alley, 900470 Constanta, Romania; 9 “St. Ap. Andrew” Emergency County Clinical Hospital, 145 Tomis Boulevard, 900591 Constanta, Romania

**Keywords:** Parkinson’s disease, exercise-induced oxygen desaturation, 6MWT, cognition, MoCA, pulmonary function, DLCO

## Abstract

**Background/Objectives**: Respiratory dysfunction in Parkinson’s disease (PD) is frequently underrecognized, particularly when resting oxygen saturation is preserved. Dynamic stress testing, however, may reveal exercise-induced oxygen desaturation, reflecting a latent functional respiratory impairment. The relationship between exertional oxygen desaturation and cognitive performance in PD remains insufficiently explored. **Objective**: To investigate the association between exercise-induced oxygen desaturation and global cognitive performance in patients with PD, and to explore the contribution of pulmonary gas exchange impairment assessed by diffusing capacity of the lung for carbon monoxide (DLCO). **Methods**: This prospective, cross-sectional, single-center observational study with consecutive enrollment included 50 patients with idiopathic Parkinson’s disease undergoing multidisciplinary respiratory evaluation following neurological assessment. Participants underwent cognitive evaluation using the Romanian version of the Montreal Cognitive Assessment (MoCA), pulmonary function testing including DLCO and total lung capacity (TLC), and a supervised 6-min walk test (6MWT) with continuous pulse oximetry. Exercise-induced oxygen desaturation was defined as a decrease in SpO_2_ of ≥4% from baseline. Correlation analyses and multivariable regression models were applied. **Results**: Exercise-induced oxygen desaturation was frequent, with 60% of patients exhibiting a ≥4% decrease in SpO_2_ during the 6MWT. Greater desaturation was significantly associated with lower MoCA scores (Spearman’s r = −0.383, *p* = 0.006). No significant associations were found between exertional desaturation and resting pulmonary function parameters, including DLCO and TLC. In multivariable analysis, lower MoCA score and levodopa–carbidopa intestinal gel treatment independently predicted greater oxygen desaturation during exercise. **Conclusions**: Exercise-induced oxygen desaturation is common in patients with PD despite preserved resting oxygenation and is associated with poorer cognitive performance. These findings suggest that exertional desaturation may reflect a dynamic functional impairment and may be associated with increased physiological vulnerability. Functional exercise testing with oxygen saturation monitoring may provide complementary information beyond resting pulmonary assessments.

## 1. Introduction

Parkinson’s disease (PD) is currently the fastest-growing neurological disorder worldwide and represents a major clinical and therapeutic challenge [1]. The disease is characterized by progressive dopaminergic neuron loss in the substantia nigra, leading to dopamine depletion and the development of cardinal motor features such as bradykinesia, rigidity, resting tremor, and postural instability [2]. As PD advances, patients experience increasing disability and the emergence of a broad spectrum of non-motor manifestations, including autonomic dysfunction, sleep disturbances, cognitive impairment, psychiatric symptoms, and respiratory abnormalities, many of which become less responsive to dopaminergic therapy and contribute substantially to disease burden and long-term complications [1,3].

In PD, respiratory involvement may have clinically significant consequences despite initially subtle symptoms. The clinical expression of respiratory dysfunction is heterogeneous and depends on the specific components of the respiratory system involved, which may allow impairment to remain underrecognized [4,5]. Alterations in ventilatory mechanics and respiratory muscle performance have been described in PD. In this context, dyspnea represents a common and clinically meaningful manifestation, frequently associated with reduced functional exercise tolerance and impaired quality of life [6,7].

Importantly, preserved oxygen saturation at rest does not exclude the clinically relevant respiratory dysfunction. During physical exertion, dynamic decreases in peripheral oxygen saturation may occur, revealing exercise-induced desaturation not captured by resting assessments [8,9]. Such exercise-induced oxygen desaturation may therefore represent a functional and clinically silent abnormality [8,9].

The use of a ≥4% decrease in peripheral oxygen saturation (SpO_2_) aligns with criteria commonly employed in sleep medicine to define clinically relevant oxygen desaturation events and their potential neurocognitive implications. Although exercise-based studies in Parkinson’s disease have not consistently detailed the rationale for this cutoff, related studies have applied oxygen desaturations exceeding 4% as indicators of hypoxic burden and have reported associations with non-motor and neuropsychiatric manifestations [10].

Cognitive dysfunction is present in a substantial proportion of patients at the time of diagnosis and represents a major risk factor for the subsequent development of dementia. The characteristic cognitive profile in PD typically involves deficits in executive function, attention, and visuospatial abilities, with relative preservation of language [11].

Emerging evidence indicates an association between respiratory physiology and cognitive performance in PD. Recent studies have shown that specific pulmonary and respiratory drive parameters correlate with global cognitive scores and disease severity across different PD motor subtypes, suggesting a potential link between impaired respiratory function and cognitive vulnerability. Nevertheless, it remains unclear whether dynamic exercise-induced oxygen desaturation, as opposed to resting pulmonary impairment, is associated with cognitive performance in PD [12].

To our knowledge, existing studies have primarily focused on resting pulmonary function or respiratory mechanics in PD, while the relationship between dynamic exercise-induced oxygen desaturation during functional walking tests and global cognitive performance remains insufficiently explored.

The aim of this study was to investigate the association between exercise-induced oxygen desaturation and cognitive performance in patients with Parkinson’s disease using a functional walking test combined with cognitive assessment. A secondary objective was to explore the potential contribution of pulmonary gas exchange impairment, assessed by DLCO, to this relationship.

We hypothesized that patients with PD who exhibit exercise-induced oxygen desaturation would demonstrate poorer cognitive performance than those without desaturation, even in the presence of normal resting oxygen saturation. In this context, the ≥4% SpO_2_ threshold was applied in the present study as a pragmatic indicator of hypoxemia-related physiological stress, in line with broader clinical and research practice rather than as a disease-specific prognostic marker.

## 2. Materials and Methods

### 2.1. Study Design and Setting

This was a prospective, single-center, observational cross-sectional study with consecutive enrollment, with all assessments performed at a single time point, conducted at the Clinical Hospital of Pneumophthisiology Constanța, a care center specialized in respiratory medicine and pulmonary rehabilitation. The study was carried out over a one-year period, from November 2024 to December 2025, and aimed to investigate the relationship between exercise-induced oxygen desaturation, as a marker of impaired respiratory reserve, and cognitive function in patients with PD.

The study protocol was reviewed and approved by the Ethics Committee of the Clinical Hospital of Pneumophthisiology Constanța, in accordance with national and international ethical standards (approval no. 5416). Prior to enrollment, all participants were informed about the study objectives, procedures, potential risks and benefits, and the voluntary nature of participation. Written informed consent was obtained from all participants before any clinical, functional, or cognitive assessment. Participant confidentiality and data protection were ensured throughout the entire study period.

### 2.2. Study Population

Patients with PD were initially evaluated by a neurologist and subsequently referred to the hospital for a comprehensive respiratory assessment as part of a multidisciplinary clinical evaluation routinely applied in this patient population. This referral reflected a systematic functional assessment approach and was not based on suspected primary pulmonary disease, allowing the study cohort to be interpreted within the context of routine clinical practice in patients with PD.

The diagnosis of PD was established prior to referral according to the Movement Disorder Society (MDS) clinical diagnostic criteria. During the study period, 50 patients were consecutively enrolled after confirmation that they met the inclusion criteria.

Eligibility for participation required:

- A confirmed diagnosis of PD;
- Preserved independent ambulation, allowing completion of the 6-min walk test (6MWT) without physical assistance;
- A cognitive and motor status sufficient to complete standardized assessments;
- The ability to understand study procedures and provide written informed consent.

Patients were excluded if they presented with any of the following conditions:

- Advanced respiratory disease (e.g., GOLD stage III–IV chronic obstructive pulmonary disease or interstitial lung disease);
- Known chronic respiratory failure or long-term oxygen therapy;
- Neurological or psychiatric disorders other than Parkinson’s disease that could interfere with testing (e.g., stroke, dementia, severe depression);
- Orthopedic or musculoskeletal disorders limiting gait performance;
- Severe visual or auditory impairment;
- Acute infection or hemodynamic instability at the time of assessment;
- Clinically significant cardiovascular disease, including heart failure NYHA functional class II–IV, reduced left ventricular ejection fraction, ischemic heart disease with functional limitation, clinically relevant arrhythmias, moderate or severe valvular heart disease, or recent acute cardiovascular events.

### 2.3. Neurological, Clinical, and Anthropometric Assessment

PD severity was clinically characterized using disease stage, while the use of device-assisted therapy (LCIG) was recorded as a treatment-related variable reflecting advanced therapeutic requirements.

Body weight and height were measured using standardized procedures, and body mass index (BMI) was calculated accordingly. Environmental pollutant exposure was assessed by structured self-report. Patients were asked about current or past occupational and environmental exposure to airborne pollutants, including dust, construction materials, chemical agents, and petroleum-derived products. Exposure was recorded as a dichotomous variable (present/absent) based on a history of sustained or repeated exposure over the working lifetime.

Additional comorbid conditions were recorded: patients with mild, stable cardiovascular comorbidities, such as well-controlled arterial hypertension or heart failure with preserved ejection fraction (NYHA functional class I), were not considered exclusion criteria.

In addition, one patient had a remote history of basal cell carcinoma treated several years prior to inclusion, with no evidence of active disease, and was therefore retained in the analysis. Age-related neuroimaging findings on brain MRI, including mild leukoaraiosis and nonspecific degenerative changes commonly observed in elderly individuals, were present in some patients. These findings were subtle in nature and were not associated with a history of stroke or transient ischemic attack, nor with imaging evidence of cerebral hypoperfusion or other clinically significant vascular pathology; therefore, they did not constitute exclusion criteria.

Other comorbidities were mainly represented by minor chronic conditions, including dermatological disorders, renal lithiasis, benign prostatic hyperplasia, and other stable medical conditions not expected to influence respiratory function, exercise performance, or cognitive assessment.

All data were entered into a secure electronic database and verified for completeness and internal consistency.

### 2.4. Cognitive Assessment

Among available cognitive screening tools, MoCA is widely used due to its high sensitivity for mild cognitive impairment and its ability to detect subtle deficits that may not identified by the Mini-Mental State Examination (MMSE) [13]. The MoCA evaluates a broad spectrum of cognitive domains, including memory, visuospatial reasoning, executive functions, attention, language and orientation. It has demonstrated good psychometric properties, with high internal consistency (Cronbach’s alpha = 0.83), supporting its reliability in both clinical and research settings [14].

In the present study, global cognitive function was assessed using the validated Romanian version of MoCA, administered under standardized conditions by trained personnel with scores adjusted for educational level according to the standard correction procedure. Total scores range from 0 to 30, with higher scores indicating better cognitive function. A cut-off score of <26 was considered indicative of mild cognitive impairment, in line with international guidelines [15]. Data were recorded immediately following completion of the test, and no assistance was provided during the assessment except for clarification of instructions when required.

### 2.5. Functional Exercise Testing and Patient Monitoring

Functional exercise capacity was evaluated using the 6MWT, performed in accordance with American Thoracic Society (ATS) guidelines [16]. Originally designed for patients with cardiopulmonary disease, it has also proven effective in neurological conditions, including PD, where it provides valuable insights into endurance and motor performance [17]. The test was conducted in an indoor 30-m corridor under continuous supervision by medical personnel experienced in functional exercise testing.

Participants were instructed to walk at their own pace for six minutes, aiming to cover the as far as possible distance, with the option to stop and rest if necessary. Standardized verbal encouragement was provided at regular intervals. The total distance walked, expressed in meters, was recorded.

SpO_2_ and heart rate were continuously monitored throughout the test using a validated portable pulse oximeter (NONIN Medical Inc., Plymouth, MN, USA). Baseline SpO_2_ was recorded after at least five minutes of seated rest, at the end of the 6MWT, and continuously throughout the test, with the lowest SpO_2_ value documented. Exercise-induced oxygen desaturation was defined as a decrease in SpO_2_ of ≥4% from baseline, consistent with commonly accepted clinical thresholds for exertional hypoxemia [9,10,18].

Perceived dyspnea and exertion were assessed immediately before and after the test using the Borg dyspnea scale and the Borg rating of perceived exertion scale, respectively.

Pulmonary function testing included assessment of DLCO, using the single-breath technique in accordance with international recommendations, with calibration and data acquisition performed on a Quark PFT system (COSMED, Rome, Italy). DLCO values were corrected for hemoglobin concentration and are expressed as a percentage of predicted values. Total lung capacity (TLC) was measured as part of static lung volume assessment and also expressed as a percentage of predicted values.

All tests were performed during the medication “on” phase. Patients were closely monitored throughout the entire procedure, and the test was interrupted if clinically significant symptoms occurred.

### 2.6. Data Analysis

All the data from the study were analyzed using IBM SPSS Statistics 25, IBM Corp., Armonk, NY, USA. and illustrated using Microsoft Office Excel/Word 2024, Microsoft Corporation, Redmond, WA, USA. Quantitative variables were tested for normal distribution using the Shapiro–Wilk Test and are reported as averages with standard deviations or medians with interquartile ranges. Quantitative independent variables with non-parametric distribution were tested between groups using the Mann–Whitney U Test/Kruskal–Wallis H Test. Correlations between quantitative variables with non-parametric distribution were calculated using Spearman’s rho correlation coefficients.

Quantitative variables with nonparametric distribution were tested between measurements using the Wilcoxon test. Quantitative independent variables with normal distributions were tested between groups using the Student T-Test/Welch T-Test (according to the equality of variances observed in the Levene Test results).

The difference in SpO_2_ evolution was predicted using univariable and multivariable (forward step-wise method) linear regression models. Models were tested for significance and goodness-of-fit. Performance of the prediction was calculated for each parameter as beta coefficients with 95% confidence intervals. To assess the robustness of the multivariable findings, sensitivity analyses were performed using alternative multivariable enter models based on clinically relevant covariates. Model assumptions were evaluated, including multicollinearity and goodness-of-fit.

Qualitative variables were written as counts or percentages and were tested between groups using Fisher’s Exact Test. The probability of high desaturation (SpO_2_ evolution difference ≥ 4%) was estimated using univariable and multivariable binomial logistic regression models. Models were tested for significance and goodness-of-fit. Performance of the prediction was calculated for each parameter as odds ratios with 95% confidence intervals.

The threshold considered for the significance level for all tests was α = 0.05.

## 3. Results

### 3.1. Characteristics of the Study Population

A total of 50 patients with PD were included in the analysis. The mean age was 70.08 ± 7.81 years, and 28 patients (56%) were male. Most patients were classified as Hoehn and Yahr stage II or III. Dyspnea was the most frequently reported respiratory symptom. Antiparkinsonian treatment included levodopa-based therapies, dopamine agonists, monoamine oxidase B inhibitors, and anticholinergic agents, frequently used in combination.

Baseline demographic, clinical, and treatment characteristics are summarized in Table 1.

### 3.2. Functional Exercise Capacity, Perceived Exertion, and Oxygen Desaturation

**Figure 1:**
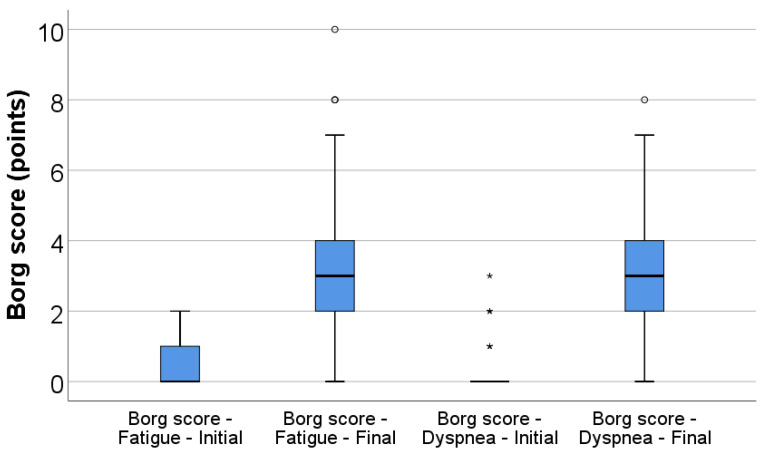
Box-plot illustration of Borg score for fatigue (initial vs. final) and dyspnea (initial vs. final). Any values that are above the 3rd quartile (75th percentile) + 1.5*interquartile range are represented as outliers represented by circles in the graph. As for values that are above the 3rd quartile (75th percentile) + 3*interquartile range, the software represents the values as extreme outliers represented by asterisk symbols in the graph.

Perceived fatigue and dyspnea increased significantly during the 6MWT. The Borg fatigue score rose from a median of 0 (IQR 0–1) at baseline to 3 (IQR 2–4) at the end of the test (*p* < 0.001), while the Borg dyspnea score increased from 0 (IQR 0–0) to 3 (IQR 2–4) (*p* < 0.001) (Table 2, Figure 1).

**Figure 2:**
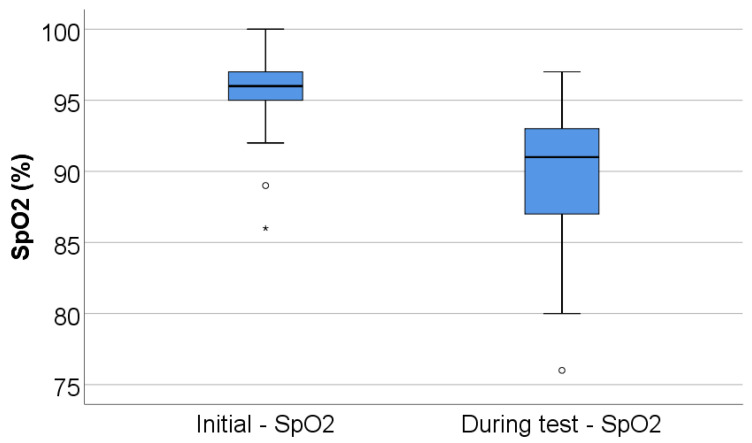
Box-plot illustration of SpO_2_ (initial vs. during test). Any values that are below the 1st quartile (25th percentile) − 1.5*interquartile range are represented as outliers represented by circles in the graph. As for values that are below the 1st quartile (25th percentile) – 3*interquartile range, the software represents the values as extreme outliers represented by asterisk symbols in the graph.

Peripheral oxygen saturation decreased significantly during exercise, from a baseline median SpO_2_ of 96% (IQR 94.75–97) to a nadir of 91% (IQR 87–93) during the test (*p* < 0.001), corresponding to a median desaturation of 5% (IQR 3–9) (Table 2, Figure 2).

### 3.3. Cognitive Performance and Pulmonary Function

The mean MoCA score was 21.06 ± 3.75 points (median 21, IQR 18.75–24). Cognitive impairment was common, with 72% of patients presenting low cognitive scores and an additional 20% presenting moderate impairment (Table 3).

Pulmonary gas exchange and lung volumes were mildly reduced at the group level. The mean DLCO was 82 ± 18.34% predicted, with 46% of patients presenting DLCO values < 80%. Mean TLC was 79.38 ± 15.71% predicted, with 52% of patients presenting reduced TLC, as presented in Table 3.

### 3.4. Association Between Oxygen Desaturation and Cognitive Performance

**Figure 3:**
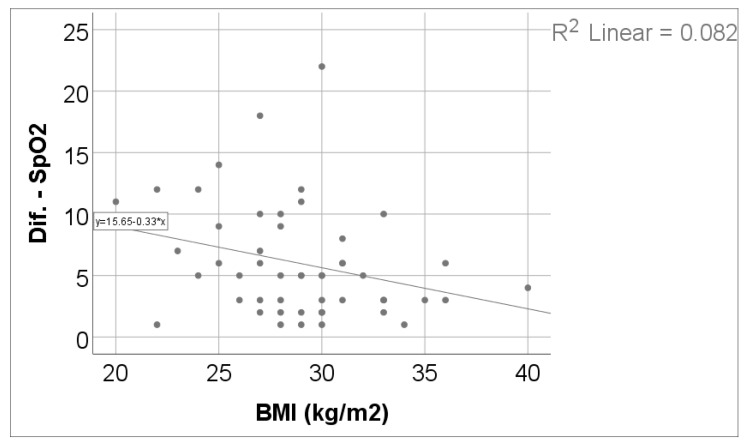
Correlation between SpO_2_ evolution difference and BMI.

**Figure 4:**
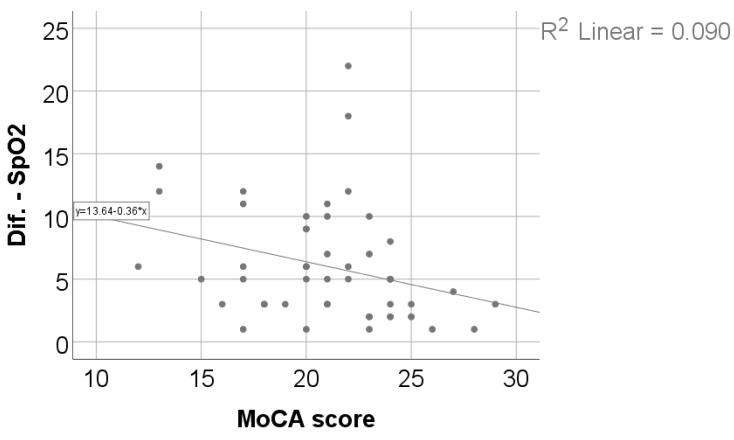
Correlation between SpO_2_ evolution difference and MoCA score.

As shown in Table 4 and Figure 3 and Figure 4, exercise-induced oxygen desaturation was significantly and negatively correlated with cognitive performance, as assessed by the MoCA score (Spearman’s r = −0.383, *p* = 0.006), with greater decreases in SpO_2_ during exercise being associated with lower MoCA scores. In the same analysis, SpO_2_ desaturation was also negatively correlated with body weight (r = −0.318, *p* = 0.024) and body mass index (r = −0.307, *p* = 0.030), while no significant associations were observed with age, 6MWT, Borg fatigue or dyspnea scores, DLCO, or TLC.

Additional correlation analyses are summarized in Supplementary Table S1.

### 3.5. Univariable and Multivariable Predictors of Cognitive Performance (MoCA Score)

To further evaluate whether exercise-induced oxygen desaturation independently explains cognitive performance, univariable and multivariable linear regression analyses were performed with MoCA score as the dependent variable.

For using PD stage as an independent predictor, a univariable generalized linear regression model was implemented. According to the results presented in the univariable models, age (*p* = 0.004), 6MWT distance (absolute—*p* < 0.001 or as percentage—*p* = 0.010), SpO_2_ difference (*p* = 0.034) and DLCO (%) (*p* = 0.034) were significant predictors, while PD stage was not significant (*p* > 0.05).

In the multivariable model, age, SpO_2_ difference, DLCO (%) and 6MWT distance (m) instead of the percentage value, due to the higher significance and being the first selection when using a forward step-wise Wald selection model (when introducing the absolute and the percentage values for 6MWT distance).

According to the results in the multivariable model, only 6MWT distance (*p* = 0.002) and SpO_2_ difference (*p* = 0.008) were significant and independent predictors for the MoCA score:

- Each increase of 1 m for the 6MWT distance significantly increases the MoCA score by 0.013 points (95% C.I. = 0.005–0.022) (*p* = 0.002);
- Each 1% increase in the decrease in SpO_2_ (SpO_2_ difference) from the initial measurement to the measurement during test significantly decreases the MoCA score by 0.257 points (95% C.I. = 0.070–0.444) (*p* = 0.008). The results are presented in Table 5.

### 3.6. Determinants of Exercise-Induced Oxygen Desaturation

As shown in Table 6, multivariable linear regression analysis identified treatment with LCIG and lower MoCA score as independent determinants of greater exercise-induced oxygen desaturation. LCIG treatment was associated with an additional decrease in SpO_2_ of 3.33 units (95% CI 0.77–5.90, *p* = 0.012), while each one-point decrease in the MoCA score was associated with a 0.36-unit increase in SpO_2_ desaturation (95% CI 0.04–0.68, *p* = 0.025).

Sensitivity analyses were conducted by sequentially excluding influential outliers identified in the multivariable linear regression models. These analyses confirmed results comparable to those obtained in the original models, with no meaningful changes in effect estimates or statistical significance (Table S2).

### 3.7. High Exercise-Induced Oxygen Desaturation (≥4%)

**Figure 5:**
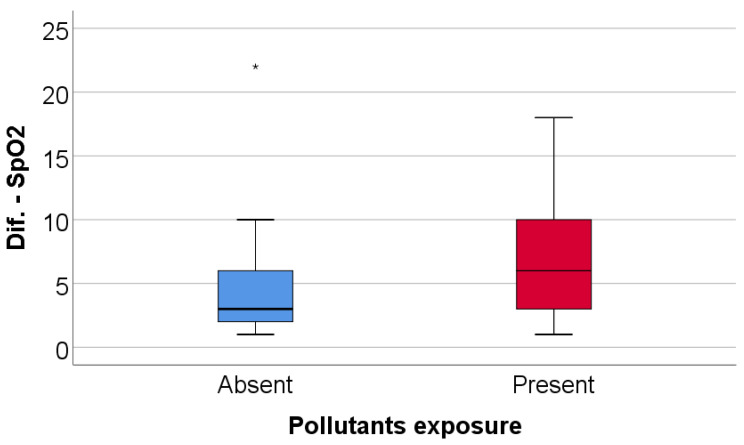
Comparison of SpO_2_ evolution difference according to the existence of pollutant exposure. Any values that are above the 3rd quartile (75th percentile) + 3*interquartile range, the software represents the values as extreme outliers represented by asterisk symbols in the graph.

Patients with high exercise-induced oxygen desaturation (≥4%) differed significantly from those without marked desaturation. Exposure to environmental pollutants was significantly more frequent among patients with high desaturation (70% vs. 40%, *p* = 0.045), as summarized in Table 7 and illustrated in Figure 5. No significant differences were observed between the two groups with respect to age, sex, Parkinson’s disease stage, smoking status, 6MWT, DLCO, or TLC.

**Figure 6:**
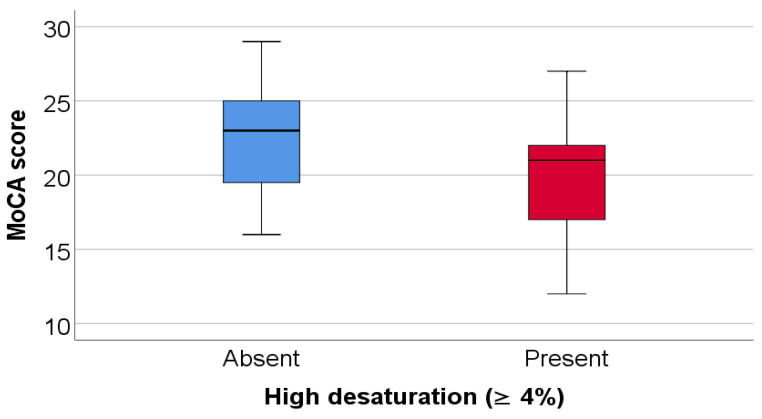
Comparison of MoCA score according to the existence of high desaturation.

As shown in Table 8 and Figure 6, patients in the high-desaturation group had significantly lower MoCA scores compared with patients without high desaturation (median 21 vs. 23, *p* = 0.035).

In univariable logistic regression analysis, both environmental pollutant exposure (OR 3.50, *p* = 0.039) and MoCA score (OR 0.82, *p* = 0.040) were significant predictors of high exercise-induced desaturation. In multivariable analysis, both variables showed a consistent direction of association, although statistical significance was not retained, likely due to the limited sample size (Table 9).

Sensitivity analyses were conducted to assess the stability of the multivariable binomial logistic regression results. After sequential exclusion of influential outliers identified in the initial models, the final model demonstrated improved fit, with both environmental pollutant exposure and MoCA score emerging as statistically significant predictors of high exercise-induced desaturation. No influential outliers were detected in the final model, and effect estimates remained consistent with those observed in the primary analysis. Results of the sensitivity analyses are summarized in Table S3.

The multivariable model shows that, in spite of the technical validity of the model, both parameters have only a tendency towards statistical significance in the same direction as in the univariable models (existence of pollutants exposure tends to increase the odds of high desaturation—*p* = 0.053, while decreasing the MoCA score tends to increase the odds of high desaturation—*p* = 0.051).

These results are determined mainly by the limited number of analyzed patients (*N* = 50); the sample size is limiting the statistical power of the multivariable model, because the introduction of both parameters in the model does not have an effect on the values of the odds ratios in comparison to the univariable model (showing the possibility of significance and independence of both predictors in a higher-performing model).

Additional graphical representations of exercise-induced oxygen desaturation according to levodopa–carbidopa intestinal gel treatment and environmental pollutant exposure are provided in the Supplementary Materials (Figures S1 and S2).

## 4. Discussion

### 4.1. Summary of the Main Findings

The main findings of this prospective observational study demonstrate that exercise-induced oxygen desaturation is significantly associated with global cognitive performance in patients with PD, even in the presence of preserved resting oxygen saturation. Greater reductions in peripheral oxygen saturation during the 6MWT were moderately and negatively correlated with MoCA scores, indicating that patients experiencing more pronounced desaturation tended to exhibit poorer cognitive performance.

In addition, the magnitude of exercise-induced oxygen desaturation was moderately and negatively associated with body weight and body mass index, suggesting that anthropometric characteristics may influence physiological responses to exertion. These findings indicate that exercise-related desaturation reflects a broader vulnerability involving both systemic and cognitive factors.

Multivariable analysis identified lower MoCA scores and treatment with levodopa–carbidopa intestinal gel as independent determinants of greater oxygen desaturation during exercise. Patients with marked desaturation (≥4%) exhibited significantly lower MoCA scores and a higher prevalence of exposure to environmental pollutants, while no significant differences were observed with respect to age, disease stage, functional exercise capacity, or resting pulmonary function parameters.

Notably, pulmonary gas exchange assessed by DLCO and static lung volumes was not significantly associated with exercise-induced desaturation or cognitive performance, underscoring the added value of functional exercise testing in identifying clinically relevant respiratory impairment not captured by resting pulmonary assessments.

### 4.2. Interpretation of the Association Between Exercise-Induced Oxygen Desaturation and Cognitive Performance

The present study demonstrates a significant association between exercise-induced oxygen desaturation and poorer cognitive performance in patients with PD, as assessed by the MoCA score. These findings suggest that oxygen desaturation occurring during functional exercise may reflect clinically relevant physiological vulnerability not apparent under resting conditions.

Previous research has shown that a substantial proportion of patients with PD exhibit reduced exercise tolerance and significant oxygen desaturation during physical exertion, even in the absence of overt pulmonary disease, indicating that dynamic stress testing can unmask respiratory impairments not detected at rest [19]. Respiratory dysfunction in PD is multifactorial, involving disturbances in central ventilatory control, respiratory muscle performance, and chest wall mechanics, which may compromise ventilatory adaptation to increased metabolic demand during exertion while remaining undetected during resting pulmonary evaluations [20,21].

In a large cross-sectional study, Li et al. demonstrated that reduced pulmonary function parameters, including peak expiratory flow, diffusing capacity, and lung volumes, were associated with poorer cognitive performance as assessed by the MoCA score, with distinct patterns observed across motor subtypes. Notably, multivariable analyses identified specific respiratory indices as independent determinants of cognitive status, suggesting that impaired respiratory physiology may contribute to cognitive vulnerability in PD. These findings indicate that respiratory dysfunction in PD extends beyond motor limitations and may be closely intertwined with non-motor manifestations, including cognitive decline [12].

Importantly, resting pulmonary function tests such as DLCO and static lung volumes are performed under standardized, low-demand conditions and may fail to capture limitations in ventilatory reserve or adaptive capacity. Accordingly, the absence of significant associations between exercise-induced oxygen desaturation and resting pulmonary parameters in the present study supports the interpretation that exertional desaturation reflects a dynamic, functional impairment rather than structural pulmonary disease [8,19]. This interpretation is further supported by longitudinal data from Kaminsky et al., who showed that respiratory impairment in Parkinson’s disease predominantly reflects a progressive reduction in lung volumes rather than intrinsic airway obstruction or parenchymal gas exchange abnormalities. In that study, declines in FEV_1_, FVC, and alveolar volume occurred despite preserved FEV_1_/FVC ratios and stable DLCO/VA, indicating a functional, restrictive pattern that may remain clinically silent under resting conditions. Taken together, these observations suggest that static pulmonary function tests may underestimate clinically relevant respiratory involvement in PD and that dynamic physiological stress—such as exercise—may be required to unmask limitations in ventilatory reserve and oxygen delivery with potential relevance for cognitive performance [22].

From a neurocognitive perspective, cognitive functions frequently affected in PD—particularly executive processes and attention—depend on distributed cortical and subcortical networks that are highly sensitive to physiological stress [11,23]. The MoCA test is especially sensitive to executive and attentional deficits in PD, domains that are vulnerable to transient physiological perturbations [24,25]. Consequently, even short-lived reductions in oxygen availability during exertion may disproportionately influence cognitive performance in individuals with diminished neurophysiological reserve [26].

Taken together, the association observed between exercise-induced oxygen desaturation and lower MoCA scores is more likely to reflect a shared vulnerability to physiological stress, in which both respiratory and cognitive systems exhibit limited compensatory capacity, rather than a direct causal relationship. This interpretation aligns with the dynamic nature of the observed impairment and with existing literature describing exertional abnormalities in PD despite preserved resting measurements [8,19].

### 4.3. Clinical Relevance and Implications for Patient Assessment

The present findings highlight the clinical relevance of assessing oxygen saturation during functional exercise in patients with PD, even when resting oxygenation and standard pulmonary function tests are within normal limits. Preserved resting SpO_2_ does not preclude the presence of clinically meaningful respiratory dysfunction, as exercise-induced desaturation may reveal limited ventilatory reserve and impaired physiological adaptation that remain undetected during static assessments.

From a practical perspective, functional exercise testing with continuous oxygen saturation monitoring, such as the 6MWT, represents a simple, low-cost, and widely accessible tool that may complement routine neurological and pulmonary evaluations. Current respiratory guidelines emphasize the value of field walking tests for identifying exertional abnormalities that are not captured by resting measurements, supporting their role in comprehensive patient assessment [8,16].

Notably, the association between exercise-induced desaturation and cognitive performance observed in this study suggests that exertional hypoxemia may serve as a marker of increased physiological vulnerability in PD. In this context, oxygen desaturation may be associated with cognitive vulnerability through several interacting physiological pathways. Intermittent or sustained hypoxemia could lead to transient reductions in cerebral oxygen delivery, potentially affecting neuronal function in brain networks commonly involved in Parkinson’s disease, particularly fronto-striatal circuits [27,28,29]. Experimental and clinical data further suggest that hypoxic stress may be accompanied by increased oxidative stress, mitochondrial dysfunction, and neuroinflammatory responses, processes that have been implicated in neurodegenerative conditions, including Parkinson’s disease [10,28,29,30].

Clinical observations also indicate that oxygen desaturation in settings such as sleep-disordered breathing has been associated with neuropsychiatric manifestations, including hallucinations, while autonomic disturbances leading to transient cerebral hypoperfusion (e.g., orthostatic hypotension) have been linked to an increased risk of cognitive impairment in Parkinson’s disease [27,28,30,31,32]. Although these associations do not establish causality, they provide a plausible physiological framework in which exercise-induced oxygen desaturation may reflect reduced neurophysiological reserve and increased susceptibility to cognitive dysfunction under conditions of physiological stress.

Identifying patients who exhibit significant desaturation during physical stress may therefore help stratify individuals at higher risk of functional and cognitive decline, even in the absence of overt respiratory symptoms.

### 4.4. Limitations of the Study

Several limitations of this study should be acknowledged. First, the relatively small sample size (*N* = 50) may have limited statistical power, particularly for multivariable analyses, and small to moderate effect sizes cannot be excluded.

Second, the cross-sectional nature of the study limits the ability to draw causal inferences regarding the relationship between exercise-induced oxygen desaturation and cognitive function and does not allow evaluation of how disease progression in PD may influence, over time, the severity of latent chronic respiratory insufficiency and cognitive performance.

Third, as this was a single-center study conducted in a specialized respiratory hospital, a potential selection effect related to the referral setting cannot be entirely excluded. However, patients were evaluated as part of a multidisciplinary clinical assessment and were not referred due to suspected primary pulmonary disease. Therefore, the findings should be interpreted within this clinical context, without assuming a specific directional impact on the observed prevalence of exercise-induced oxygen desaturation or the strength of associations.

Fourth, exercise-induced hypoxemia was assessed using pulse oximetry rather than arterial blood gas analysis; although continuous SpO_2_ monitoring during the 6MWT is widely accepted in both clinical practice and research settings, a certain degree of measurement variability cannot be entirely excluded.

Finally, although pulmonary gas exchange and lung volumes were assessed using DLCO and TLC, not all potentially relevant clinical or behavioral variables were available for inclusion in the statistical models. Factors such as educational attainment, affective symptoms, or habitual physical activity may contribute to interindividual variability in cognitive performance and exercise-related oxygenation. To address this, multivariable models and sensitivity analyses were applied, which consistently supported the observed associations.

Taken together, these limitations should be acknowledged when interpreting the present findings.

### 4.5. Future Directions

Future studies should adopt longitudinal designs to clarify the temporal relationship between exercise-induced oxygen desaturation and cognitive performance in patients with PD. Particular attention should be directed toward individuals with preserved resting oxygen saturation who nevertheless develop significant desaturation during exertion, as this silent functional impairment may remain clinically unrecognized and potentially progress over time. Repeated longitudinal assessments of exercise oxygenation, pulmonary function, and cognitive status may help determine whether worsening exertional respiratory dysfunction parallels cognitive decline.

In addition, future research should evaluate whether systematic screening strategies incorporating simple exercise-based respiratory tests, such as field walking assessments with continuous oxygen monitoring, can improve early identification of patients at risk. In those exhibiting marked exertional hypoxemia—particularly when oxygen saturation falls below clinically relevant thresholds such as 90% during sustained activity—the potential role of portable supplemental oxygen during prolonged or repetitive exertion warrants investigation under controlled conditions.

Finally, interventional studies assessing targeted respiratory training or pulmonary rehabilitation programs are needed to determine whether improving exercise-related oxygenation translates into meaningful benefits in functional capacity, symptom burden, and cognitive outcomes in patients with PD.

## 5. Conclusions

In conclusion, the present study suggests that exercise-induced oxygen desaturation during 6MWT may be associated with poorer cognitive performance in patients with PD, even in the presence of preserved resting oxygen saturation. In contrast, no significant relationship was observed between exertional desaturation and resting pulmonary function parameters, including DLCO and TLC.

These findings indicate that respiratory impairment in PD may not be fully captured by resting pulmonary assessments and may become evident only under conditions of increased physiological demand. Exercise-induced oxygen desaturation may therefore reflect reduced respiratory or systemic reserve rather than overt baseline pulmonary impairment.

Although causality cannot be inferred from the current data, the observed associations highlight the potential relevance of dynamic oxygenation assessment in the functional evaluation of patients with Parkinson’s disease. Further longitudinal and mechanistic studies are warranted to clarify the clinical significance and underlying pathways linking exertional hypoxemia and cognitive vulnerability in this population.

## Figures and Tables

**Table 1:** Characteristics of the analyzed patients.

Parameter	Value
Age (Mean ± SD, Median (IQR))	70.08 ± 7.81, 71 (65.75–75)
Gender (Male) (Nr., %)	28 (56%)
Height (Mean ± SD, Median (IQR))	166.62 ± 9.44, 166 (159.5–175)
Weight (Mean ± SD, Median (IQR))	80.26 ± 15.06, 80 (70–90)
BMI (Mean ± SD, Median (IQR))	28.9 ± 3.87, 29 (27–31)
Medical history (Nr., %)	
Comorbidities	45 (90%)
Essential hypertension	28 (56%)
Cardiovascular diseases	20 (40%)
Metabolic diseases (Diabetes)	16 (32%)
Neurovascular diseases	13 (26%)
Neoplasia	1 (2%)
Depression	7 (14%)
Gastrointestinal diseases	3 (6%)
Other conditions	4 (8%)
Parkinson’s disease staging	
Stage 1	3 (6%)
Stage 2	25 (50%)
Stage 3	21 (42%)
Stage 4	1 (2%)
Smoking	
Non-smoker	24 (48%)
Active smoker	9 (18%)
Ex-smoker	17 (34%)
Packages/year (Mean ± SD, Median (IQR))	25.08 ± 20.52, 22.5 (8.75–31.25)
Pollutants exposure (Nr., %)	29 (58%)
Treatment (Nr., %)	
Levodopa	9 (18%)
Levodopa–Carbidopa intestinal gel	15 (30%)
Pramipexol	22 (44%)
Rasagiline	9 (18%)
Trihexyphenidyl	2 (4%)
Levodopa–carbidopa	12 (24%)
Selegiline	2 (4%)
Symptoms (Nr., %)	
Dyspnea	45 (90%)
Coughing	23 (46%)
Chest constriction	12 (24%)

**Table 2:** Evolution of Borg scores and SpO_2_.

Borg Score—Fatigue			
Measurement	Mean ± SD	Median (IQR)	*p* *
Initial	0.48 ± 0.84	0 (0–1)	<0.001
Final	3.6 ± 2.02	3 (2–4)	
Dif.	3.12 ± 2.03	3 (2–4)	-
Borg Score—Dyspnea			
Measurement	Mean ± SD	Median (IQR)	*p* *
Initial	0.24 ± 0.65	0 (0–0)	<0.001
Final	2.94 ± 1.76	3 (2–4)	
Dif.	2.7 ± 1.63	2.5 (1–4)	-
SpO_2_			
Measurement	Mean ± SD	Median (IQR)	*p* *
Initial	95.72 ± 2.45	96 (94.75–97)	<0.001
During test	89.72 ± 4.71	91 (87–93)	
Dif.	6 ± 4.53	5 (3–9)	-

* Related-Samples Wilcoxon Signed Rank Test.

**Table 3:** Performance analyzed characteristics of the patients in the study.

Parameter	Value
Travel distance (m) (Mean ± SD, Median (IQR))	335.26 ± 111.85, 338 (249–435)
Travel distance (%) (Mean ± SD, Median (IQR))	71.88 ± 23.32, 71.1 (55.9–83.2)
MoCA score (Mean ± SD, Median (IQR))	21.06 ± 3.75, 21 (18.75–24)
MoCA score category (Nr., %)	
Normal—26–30 points	4 (8%)
Low—25–18 points	36 (72%)
Moderate—10–17 points	10 (20%)
DLCO% (Mean ± SD, Median (IQR))	82 ± 18.34, 80.5 (68.75–91.75)
Low DLCO% (<80%) (Nr., %)	23 (46%)
TLC% (Mean ± SD, Median (IQR))	79.38 ± 15.71, 77 (68.75–90)
Low TLC% (<80%) (Nr., %)	26 (52%)

**Table 4:** Correlations of SpO_2_ evolution differences and other analyzed characteristics.

Correlation	*p* *
SpO_2_ Dif. × Age	0.733, R = −0.049
SpO_2_ Dif. × Height	0.537, R = −0.089
SpO_2_ Dif. × Weight	0.024, R = −0.318
SpO_2_ Dif. × BMI	0.030, R = −0.307
SpO_2_ Dif. × Packages/year	0.929, R = 0.018
SpO_2_ Dif. × Travel distance (m)	0.836, R = −0.030
SpO_2_ Dif. × Travel distance (%)	0.919, R = −0.015
SpO_2_ Dif. × Borg score—Fatigue—Initial	0.214, R = −0.179
SpO_2_ Dif. × Borg score—Fatigue—Dif.	0.571, R = 0.082
SpO_2_ Dif. × Borg score—Dyspnea—Initial	0.441, R = 0.111
SpO_2_ Dif. × Borg score—Dyspnea—Dif.	0.363, R = 0.131
SpO_2_ Dif. × MoCA score	0.006, R = −0.383
SpO_2_ Dif. × DLCO%	0.242, R = −0.169
SpO_2_ Dif. × TLC	0.436, R = 0.113

* Spearman’s rho Correlation Coefficient.

**Table 5:** Univariable and multivariable linear regression models used in the prediction of MoCA score.

Parameter	Univariable		Multivariable		
	B (95% C.I.)	*p*	B (95% C.I.)	*p*	VIF
Age	−0.191 (−0.319–−0.063)	0.004	−0.086 (−0.208–0.036)	0.162	1.312
PD stage *					
Stage I (Ref.)	-	-	-	-	
Stage II	−2.000 (−6.372–2.372)	0.370	-	-	
Stage III	−2.286 (−6.702–2.130)	0.310	-	-	
Stage IV	1.000 (−7.262–9.262)	0.812	-	-	
6MWT distance (m)	0.017 (0.008–0.025)	<0.001	0.013 (0.005–0.022)	0.002	1.249
6MWT distance (%)	0.058 (0.015–0.102)	0.010	-	-	
SpO_2_ difference	−0.248 (−0.477–−0.019)	0.034	−0.257 (−0.444–−0.070)	0.008	1.042
DLCO (%)	0.083 (0.028–0.137)	0.004	0.046 (−0.003–0.094)	0.063	1.140

* Univariable generalized linear regression model. Multivariable linear regression model: Adjusted R^2^ = 0.405, Durbin–Watson test score = 2.009, F (4, 45) = 9.343, *p* < 0.001.

**Table 6:** Univariable and multivariable linear regression models used in the prediction of SpO_2_ evolution difference.

Parameter	Univariable		Multivariable *	
	B (95% C.I.)	*p*	B (95% C.I.)	*p*
Exposure	1.97 (−0.60–4.54)	0.131	-	-
Levodopa–Carbidopa intestinal gel	3.33 (0.66–6.00)	0.016	3.33 (0.77–5.90)	0.012
Weight	−0.066 (−0.15–0.019)	0.125	-	-
BMI	−0.33 (−0.66–−0.009)	0.044	-	-
MoCA score	−0.36 (−0.69–−0.02)	0.034	−0.36 (−0.68–−0.04)	0.025

* Forward Step-Wise Method.

**Table 7:** Distribution of the patients according to the existence of high desaturation (≥4%) and analyzed characteristics.

Parameter (Nr., %)	Absent	Present	*p* *
Gender (Male)	12 (60%)	16 (53.3%)	0.773
Medical history	18 (90%)	27 (90%)	1.000
Essential HT	10 (50%)	18 (60%)	0.567
Cardiovascular	8 (40%)	12 (40%)	1.000
Metabolic	5 (25%)	11 (36.7%)	0.538
Neurovascular	6 (30%)	7 (23.3%)	0.744
Neoplasia	1 (5%)	0 (0%)	0.400
Depression	5 (25%)	2 (6.7%)	0.100
Gastrointestinal	0 (0%)	3 (10%)	0.265
Other conditions	1 (5%)	3 (10%)	0.641
PD stage	Absent	Present	*p* *
Stage 1	1 (5%)	2 (6.7%)	0.881
Stage 2	9 (45%)	16 (53.3%)	
Stage 3	10 (50%)	11 (36.7%)	
Stage 4	0 (0%)	1 (3.3%)	
Smoking	Absent	Present	*p* *
Non-smokers	11 (55%)	13 (43.3%)	0.596
Active smokers	4 (20%)	5 (16.7%)	
Ex-smokers	5 (25%)	12 (40%)	
Exposure	8 (40%)	21 (70%)	0.045
Levodopa	6 (30%)	3 (10%)	0.130
Levodopa–Carbidopa intestinal gel	4 (20%)	11 (36.7%)	0.345
Pramipexole	9 (45%)	13 (43.3%)	1.000
Rasagiline	4 (20%)	5 (16.7%)	1.000
Trihexyphenidyl	1 (5%)	1 (3.3%)	1.000
Levodopa–carbidopa	5 (25%)	7 (23.3%)	1.000
Selegiline	0 (0%)	2 (6.7%)	0.510
Dyspnea	17 (85%)	28 (93.3%)	0.377
Coughing	11 (55%)	12 (40%)	0.388
Chest constriction	3 (15%)	9 (30%)	0.317

* Fisher’s Exact Test.

**Table 8:** Comparison of other important characteristics according to the existence of high desaturation in evolution (≥4%).

Parameter (Median (IQR))	Absent	Present	*p* *
Age (Mean ± SD)	70.05 ± 8.58	70.1 ± 7.41	0.983 **
Height (Mean ± SD)	167.45 ± 10.15	166.07 ± 9.08	0.617 **
Weight (Mean ± SD)	83.85 ± 13.15	77.87 ± 15.97	0.171 **
BMI (Mean ± SD)	29.95 ± 3.38	28.2 ± 4.08	0.119 **
Packages/year	25 (7.5–35)	20 (12.5–32.5)	1.000
Travel distance (m) (Mean ± SD)	349.35 ± 122.73	325.87 ± 105.08	0.473 **
Travel distance (%) (Mean ± SD)	74.34 ± 23.6	70.25 ± 23.4	0.549 **
Borg score—Fatigue—initial	0 (0–2)	0 (0–0)	0.092
Borg score—Fatigue—Dif.	2.5 (2–4)	3 (2–4.25)	0.731
Borg score—Dyspnea—initial	0 (0–0)	0 (0–0)	0.818
Borg score—Dyspnea—Dif.	2 (1–4)	3 (2–4)	0.386
MoCA score	23 (19.25–25)	21 (17–22.25)	0.035
DLCO% (Mean ± SD)	81.85 ± 13.88	82.1 ± 21.03	0.960 ***
TLC% (Mean ± SD)	76.35 ± 12.47	81.4 ± 17.46	0.270 **

* Mann–Whitney U Test, ** Student T-Test, *** Welch T-Test.

**Table 9:** Univariable and multivariable binomial logistic regression models used for the prediction of the existence of high desaturation (≥4%).

Parameter	Univariable		Multivariable *	
	OR (95% C.I.)	*p*	OR (95% C.I.)	*p*
Exposure	3.50 (1.06–11.47)	0.039	3.40 (0.98–11.82)	0.053
MoCA score	0.82 (0.68–0.99)	0.040	0.82 (0.68–1.001)	0.051

* Multivariable enter model, χ^2^ (2) = 8.899, *p* = 0.012, Nagelkerke R^2^ = 0.220, Hosmer and Lemeshow Test—*p* = 0.179, Overall accuracy = 64%. *N* = 20/*N* = 30 (Low desaturation/High desaturation).

## Data Availability

The datasets generated and analyzed during the current study are not publicly available due to ethical restrictions and patient confidentiality but are available from the corresponding author on reasonable request.

## Supplementary Materials

The following supporting information can be downloaded at: https://www.mdpi.com/article/10.3390/jcm15020899/s1. Figure S1: Comparison of SpO_2_ evolution difference according to the existence of treatment with Levodopa–Carbidopa gel; Figure S2: Distribution of the patients according to pollutant exposure and existence of high desaturation in evolution (≥4%); Table S1: Comparison of SpO_2_ evolution difference across analyzed characteristics; Table S2: Multivariable linear regression model used in the prediction of SpO_2_ difference; Table S3: Univariable and multivariable binomial logistic regression models used for the prediction of the existence of high desaturation (≥4%).
