# Supplementary material for: Exercise-Induced Oxygen Desaturation and Cognitive Performance in Patients with Parkinson’s Disease: A Prospective Observational Study
Source: J Clin Med. 2026 Jan 22;15(2):899. doi: 10.3390/jcm15020899 (PMC12842213; doi:10.3390/jcm15020899)
Supplement: Supplementary file 1 [file jcm-15-00899-s001.zip › jcm-4091281-supplementary.pdf]

Table S1. Comparison of SpO2 evolution difference across analyzed characteristics

SpO2 - Median (IQR)	Male		Female		p*
**Gender**	5 (3-6)		6.5 (2.75-11)		0.288
Medical history	**Absent**		**Present**		**p***
**Comorbidites**	5 (2-14.5)		5 (3-8.5)		0.826
**Essential hypertension**	5 (2-6.75)		5.5 (3-10)		0.234
**Cardiovascular**	5 (3-9.25)		5 (2.25-6.75)		0.536
**Metabolic**	5 (2.75-10)		5.5 (3-7)		0.722
**Neurovascular**	5 (3-10)		5 (1.5-5.5)		0.114
**Depression**	5 (3-10)		3 (1-5)		0.060
**Gastrointestinal**	5 (3-8.5)		7 (6-9.5)		0.233
**Other**	5 (2.75-9)		6.5 (3.5-11)		0.436
**PD stage**	**Stage 1**	**Stage 2**	**Stage 3**	**Stage 4**	**p****
	5 (3.5-11.5)	6 (3-8)	4 (2-10)	5	0.882
**Smoking**	**Non-smoker**	**Smoker**		**Ex-smoker**	**p****
	5 (2-9.5)	5 (3-8)		5 (3-6)	0.865
**Pollutants exposure**	**Absent**		**Present**		**p***
	3 (2-6.5)		6 (3-10.5)		**0.035**
Treatment	**Absent**		**Present**		**p***
**Levodopa**	5 (3-9)		3 (2-7.5)		0.176
**Lecigon**	5 (2-6)		9 (3-12)		**0.043**
**Oprymea**	5 (2.25-11)		5 (3-6)		0.330
**Rasagiline**	5 (3-9)		5 (1.5-10)		0.747
**Trihexifin**	5 (3-9)		4.5 (3-6)		0.901
**Isicom**	5 (2.75-10)		5 (3-6)		0.443
**Seleges**	5 (3-9)		5 (5-5)		1.000
Symptoms	**Absent**		**Present**		**p***
**Dyspnea**	3 (1.5-9)		5 (3-9)		0.488
**Coughing**	5 (3-11)		4 (2-6)		0.139
**Chest constriction**	5 (3-7.25)		5.5 (2-11.5)		0.450

***Mann-Whitney U Test, **Kruskal-Wallis H Test**

Table S2. Multivariable linear regression model used in the prediction of SpO2 difference

Parameter	B (95% C.I.)	p	VIF
**LCIG**	2.405 (0.467-4.343)	0.016	1.049

**MoCA score**	**-0.354 (-0.590 - -0.118)**	**0.004**	**1.076**
**BMI**	-0.180 (-0.414 – 0.054)	0.127	1.126

Multivariable linear regression model: Adjusted R^2 = 0.305, Durbin-Watson test score = 2.212, F (3,44) = 7.888, p<0.001

Table S3. Univariable and multivariable binomial logistic regression models used for the prediction of the existence of high desaturation (≥ 4%)

Parameter	Univariable		Multivariable*	
	OR (95% C.I.)	p	OR (95% C.I.)	p
**Exposure**	4.500 (1.305-15.515)	**0.017**	5.928 (1.416-24.816)	**0.015**
**MoCA score**	0.755 (0.604-0.943)	**0.013**	0.719 (0.559-0.924)	**0.010**

***Multivariable enter model, χ^2 (2) = 15.046, p=0.001, Nagelkerke R^2 = 0.364, Hosmer and Lemeshow Test – p=0.136, Overall accuracy = 68.8%. N= 19/N=29 (Low desaturation/High desaturation).**

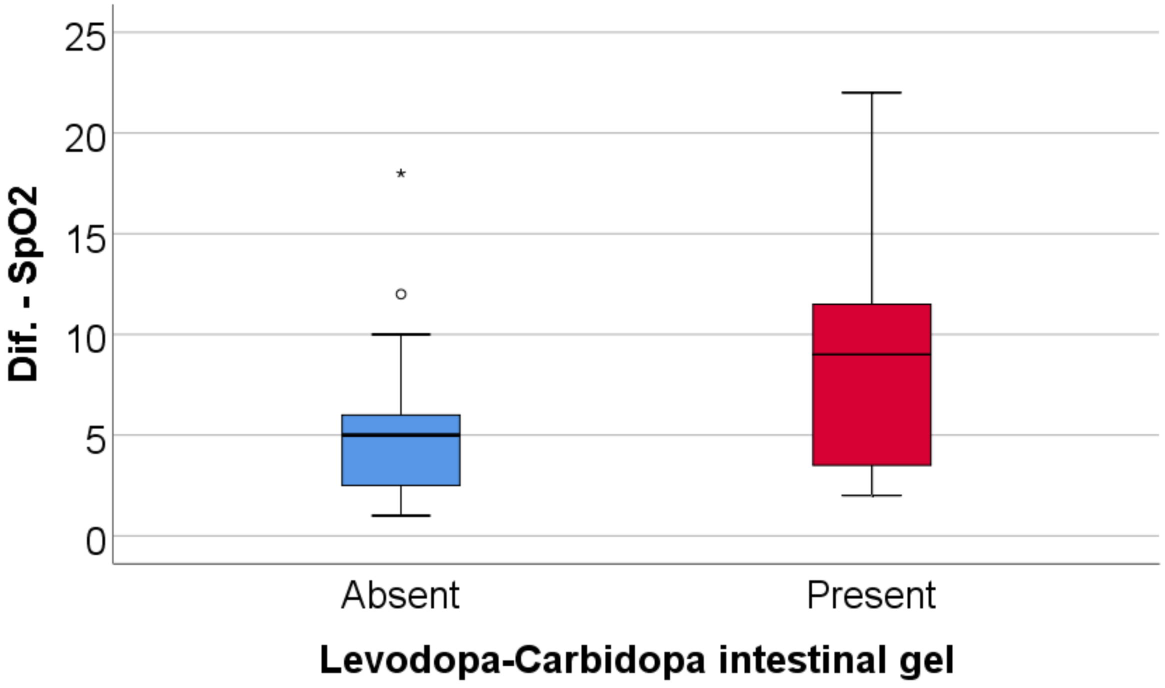

Figure S1. Comparison of SpO2 evolution difference according to the existence of treatment with Levodopa-Carbidopa gel

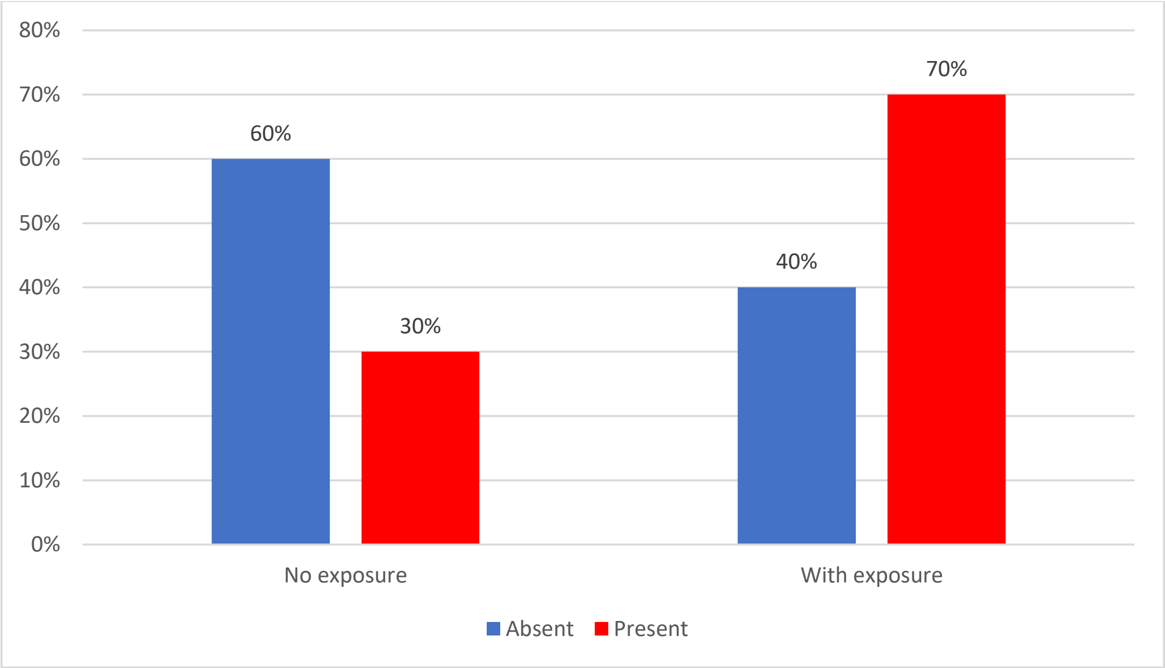

Figure S2. Distribution of the patients according to pollutants exposure and existence of high desaturation in evolution (≥ 4%)
